# Mesenchymal stem cells in inflammatory microenvironment potently promote metastatic growth of cholangiocarcinoma *via* activating Akt/NF-κB signaling by paracrine CCL5

**DOI:** 10.18632/oncotarget.17793

**Published:** 2017-05-11

**Authors:** Wei Zhong, Yinping Tong, Yang Li, Jiahui Yuan, Shaoping Hu, Tianhui Hu, Gang Song

**Affiliations:** ^1^ Cancer Research Center, Medical College of Xiamen University, Xiamen 361102, China; ^2^ Department of General Surgery, The Affiliated Southeast Hospital of Xiamen University, Zhangzhou 363000, China

**Keywords:** cholangiocarcinoma, mesenchymal stem cell, inflammatory microenvironment, CCL5

## Abstract

Our previous work has demonstrated that mesenchymal stem cells (MSCs) could induce metastatic growth of the inflammation-related cholangiocarcinoma (CCA). However, the functional mechanism of MSCs on CCA progression in the early inflammatory microenvironment remained undetermined. Here, we showed that TNF-α and IFN-γ-induced inflammatory microenvironment stimulated the expression of TNF-α, CCL5, IL-6, IDO, and activated the NF-κB signaling with p65 nuclear translocation in MSCs cells. CCA cell lines QBC939 and Mz-chA-1 exposed to the conditioned medium of MSCs after being stimulated by TNF-α and IFN-γ (TI-CM) exhibited enhanced mobility. Moreover, MSCs pre-stimulated by both inflammatory cytokines (TI-MSCs) increased tumor metastasis *in vivo*. The conditioned medium of TI-MSCs stimulated the transcription of *snail*, *slug*, *ZEB1* and *ZEB2*. Next, the expression of CCL5 of TI-MSCs was verified by ELISA, which indicated that MSCs contributed to CCA migration and metastasis in a paracrine fashion. CCA cells treated with TI-CM up-regulated CCA chemokine receptors, especially CCR5; CCL5 neutralizing antibody or CCR5 inhibitor Maraviroc inhibited the effects of MSCs on CCA cells migration. We also found that Akt/NF-κB signaling was activated by CCL5/CCR5 axis, which increased the expression of MMP2, MMP9. Together, these findings suggest that MSCs in tumor inflammatory microenvironment are elicited of CCL5, which activate AKT/NF-κB signaling and lead to metastatic growth of CCA cells.

## INTRODUCTION

Cholangiocarcinoma (CCA) is the second most common primary hepatobiliary cancer, after hepatocellular cancer [1]. Affected by many factors, especially primary sclerosing cholangitis, the incidence of CCA has a rising tendency [2, 3]. Between 1973 and 2012, the reported U.S. incidence of intrahepatic cholangiocarcinoma (ICC) increased from 0.44 to 1.18 cases per 100,000 [4]. In inflammatory microenvironment, cholangiocytes may undergo malignant transformation, which makes cholangiocytes more likely to form CCA. Most CCA has the characteristics of insidious early and atypical clinical symptom, rapid progression, high malignant degree and poor prognosis. Michael et al. performed a Meta-analysis including 57 studies showed that median 5-year overall survival (OS) of ICC was approximately 30% [5]. The recurrence and metastasis of CCA has become the key problem for patients to get good treatment effects and achieve long-term survival. Therefore, exploring the mechanism of recurrent and metastasis, and finding effective treatments are of great value.

Mesenchymal stem cells (MSCs) are multipotent adult progenitor cells, which have attracted much attention due to their multi-differentiation potential, low immunogenicity, as well as strong tropism to wounds, inflammatory sites and tumor sites specifically [6]. They also have the ability to regulate the secretion of cytokines in immune response cells, and induce a more anti-inflammatory and tolerant environment [7, 8]. However, culture conditions have striking effects on the phenotype and function of MSCs [9]. There were reports that inflammatory condition was a major activator of the immunosuppressive capacity of MSCs [10]. MSCs could increase colon cancer growth; the growth-promoting effect was further accelerated when the MSCs were pre-stimulated by inflammatory cytokines TNF-α and IFN-γ [11]. This indicates that, MSCs, as an important component, play an important role in inflammatory tumor microenvironment.

MSCs may activate a series of tumor signaling pathways through the release of cytokines to influence the development of tumor cells, these signaling pathways may increase or inhibit tumor growth and metastasis, or leading to cancer cell apoptosis [12]. MSCs have a promoting metastasis potential to many tumors, such as breast cancer [13, 14], osteosarcoma [15], melanoma [16], colon cancer [17]. Our previous work had also demonstrated that MSCs could effectively induce metastatic growth and chemoresistance of CCA *via* activation of *Wnt/*β-*catenin* signaling pathway [18]. On the contrary, studies also showed that MSCs can inhibit the growth and migration of glioma cells *via* down-regulating the PI3K/Akt pathway [19]. Hence, there are many unknown factors and risks for the application of MSCs, and further study of the effect of MSCs on tumor is quite important.

CCL5 belongs to the chemokine family, and it is widely established as an inflammatory chemokine secreted by many cell types including T lymphocytes, macrophages, platelets, and certain types of tumor cells [20, 21]. The activity of CCL5 is mediated through binding to CCR1, CCR3 and mainly CCR5 [20]. It has been demonstrated that the release of CCL5 by cells of the tumor microenvironment promotes the liver metastasis of breast cancer cells [22]. In the breast cancer microenvironment, cancer cell stimulate de novo secretion of CCL5 from MSCs, and CCL5 acts in a paracrine fashion to enhance cancer cell migration, invasion and metastasis [13]. CCL5 secreted from MSCs can promote the migration and invasion of Huh7 cells via PI3K/AKT signal pathway, and may be an important factor in hepatocellular carcinoma metastasis [23]. In addition, the effects of CCL5 on gastric, ovarian, and prostate cancer occurrence and metastasis were widely studied [20], while the mechanisms of CCL5 expressed by MSCs on CCA cells migration and invasion are poorly understood.

In this study, TNF-α and IFN-γ were used to simulate inflammatory microenvironment *in vitro*, and a high expression of CCL5 in MSCs was found under this condition. Then we investigated the functional mechanisms of MSCs being stimulated by inflammatory cytokines on CCA cells migration and metastasis *in vitro* and *in vivo* models*.* Our studies showed that MSCs in tumor inflammatory microenvironment secrete a number of chemokines and demonstrate that CCL5 plays a significant role in the migration of the CCA cells *via* CCR5, which induce AKT/NF-κB signaling activation of CCA cells that lead to metastatic growth.

## RESULTS

### Pro-inflammatory cytokines increase the migration and cytokines expression in MSCs

**Figure 1:**
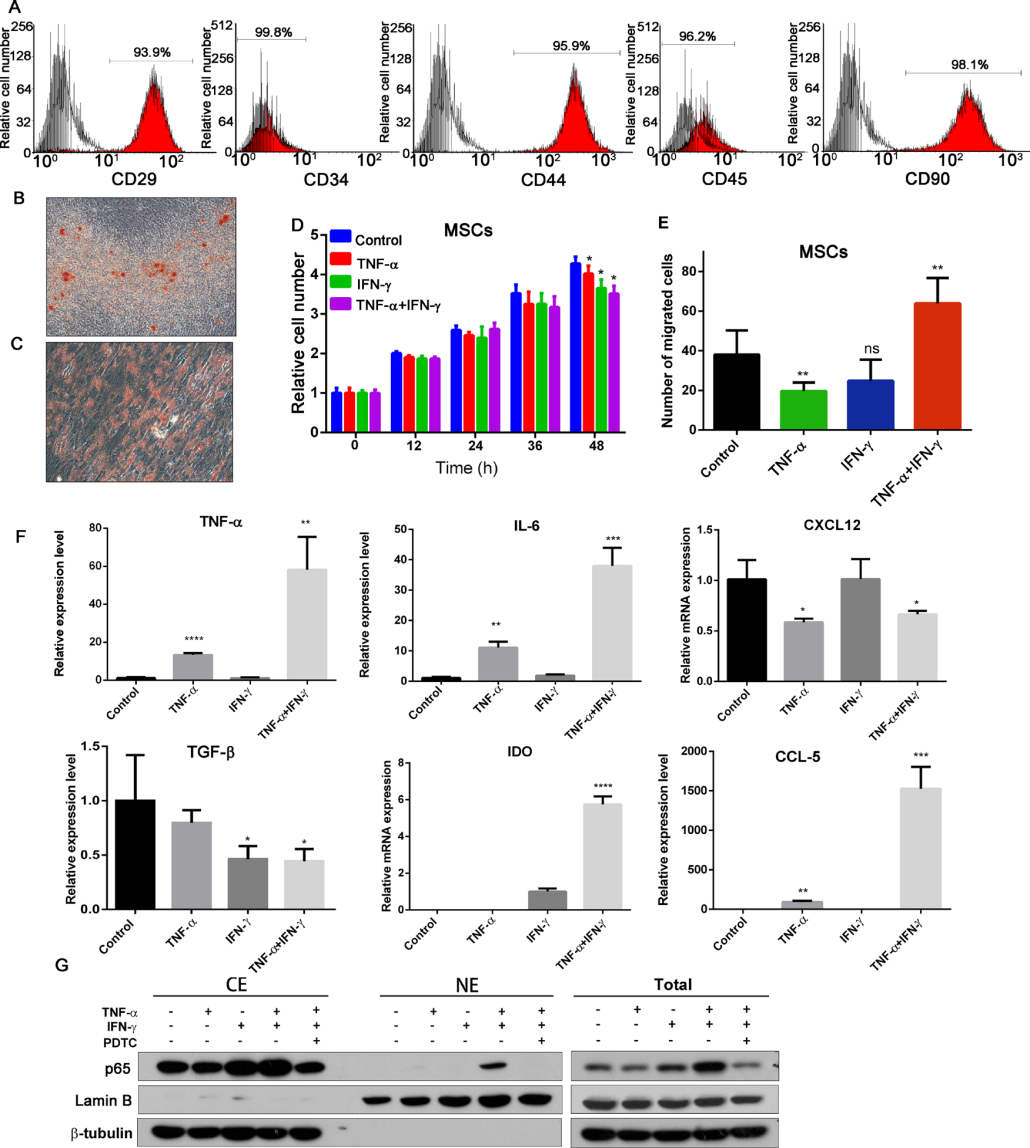
Effects of inflammatory cytokines on MSCs **(A)** Human umbilical cord-derived MSCs were characterized by flow cytometry assay with CD29, CD34, CD44, CD45 and CD90 antibodies, and the IgG1 and IgG2b are the isotype. (**B**) Oil Red O staining of adipogenic differentiated hUC-MSCs (200×). (**C**) Alizarin Red S staining of osteogenic differentiated hUC-MSCs (200×). (**D**) Effects of inflammatory factors on cell viability of MSCs; (**E**) Effects of inflammatory environment on migration ability of MSCs. (**F**) The expression of *TNF-α, IL-6, TGF-β*, *CXCL12, IDO and CCL5,* in each group of MSCs. Date were expressed as fold change (means ± S.D.) over controls, *p < 0.05, **p < 0.01; two-tailed Student’s *t* test . (**G**) TNF-α and IFN-γ pretreated MSCs increased the p65 expression and nuclear translocation. Abbreviations: PTDC: pyrrolidine dithiocarbamate.

Firstly, Human umbilical cord mesenchymal stem cells (hUC-MSCs) were identified by flow cytometry assay (Figure 1A) and induced differentiation assay (Figure 1B and 1C) as previously described [18]. To investigate whether the inflammatory environment could affect MSCs proliferation, migration and cytokines secretion, we examined MSCs stimulated by both TNF-α (20 ng/ml) and IFN-γ (50 ng/ml) on cell viability by MTT assay, migration by transwell assay, and cytokines expression level by real-time PCR analysis. As shown in Figure 1D, inflammatory cytokines has a slight reduce effect on MSCs proliferation during the 2 days period. The ability of cell migration was significantly increased when MSCs were treated with TNF-α and IFN-γ (Figure 1E). This may be one of the reasons for MSCs targeting inflammation and tumor sites.

MSCs could secrete various cytokines to mediate their immune-modulator actions, and affect tumorigenicity, cancer cell proliferation and metastasis. So we detected the *TNF-α, IL-6, TGF-β, CXCL12, IDO* and *CCL5* mRNA transcription in MSCs after treated with TNF-α and/or IFN-γ for 6 hours (Figure 1F). These results showed that combination of TNF-α and IFN-γ could increase *TNF-α, IL-6, CCL5, IDO* transcription, while decrease *TGF-β* and *CXCL12*. We also investigated the NF-κB P65 expression and translocation after MSCs treated by the inflammatory cytokines (Figure 1G). The expression of P65 was increased significantly, and translocate to nuclei. PDTC is an inhibitor of NF-κB; it can inhibit the TNF-α and IFN-γ induced NF-κB activation on MSCs. Consequently, inflammatory cytokines TNF-α and IFN-γ could promote MSCs migration, activate NF-κB signal pathway and increase the cytokines and chemokines expression.

### MSCs pretreated by pro-inflammatory cytokines increase the migration and metastasis of CCA cells

**Figure 2:**
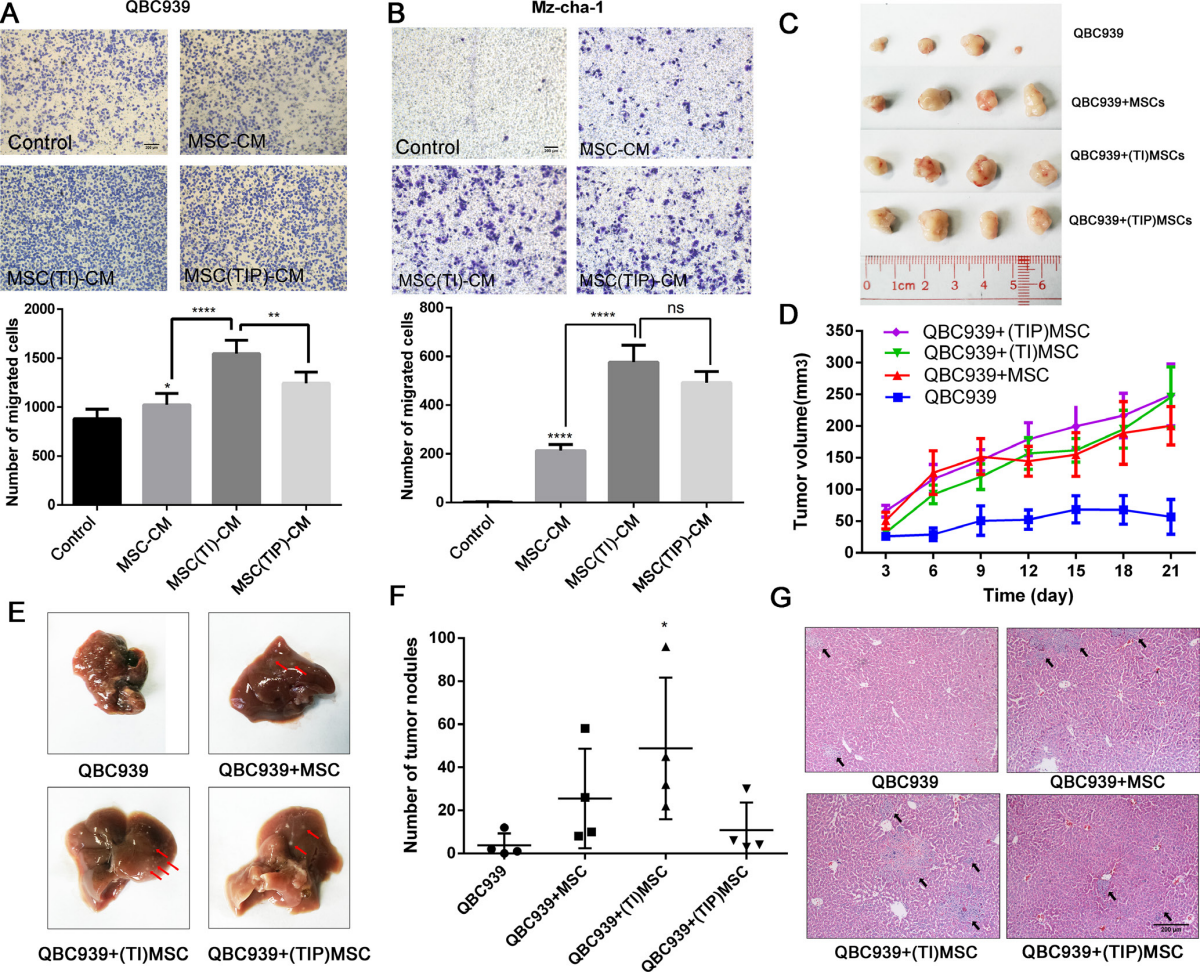
MSCs treated with inflammatory factors increase the CCA cells migration and metastasis MSCs in inflammatory environment increase the migration ability of QBC939 (**A**) and Mz-chA-1 (**B**). MSC-CM: MSCs conditioned medium. MSC(TI)-CM: conditioned medium of MSCs pretreated with TNF-α and IFN-γ. MSC(TIP)-CM: conditioned medium of MSCs pretreated with TNF-α, IFN-γ and PDTC. (**C**) The image of xenograft tumor from each group (*n =* 4). (**D**) Tumor volume measurements of each group. (**E**) Representative gross image of mouse livers from each group. The black arrow indicate the tumor nodules. (**F**) The number of tumor nodules metastasize to livers. (**G**) Representative histology image of H&E staining of liver sections. *P < 0.05, **P < 0.01, ****P < 0.0001, ns: not significant; two-tailed Student’s *t* test. Abbreviations: PTDC: pyrrolidine dithiocarbamate.

We next examined the effects of MSCs stimulated by both TNF-α and IFN-γ on cell migration using transwell migration assay. The conditioned medium from MSCs pretreated by TNF-α and IFN-γ was gathered and used for cancer cell migration assay. MSC-CM represent the conditioned medium of MSCs, MSC(TI)-CM represent the medium from MSCs pretreated by TNF-α and IFN-γ, and MSC(TIP)-CM represent the medium from MSCs treated by TNF-α, IFN-γ and PDTC. As shown in Figure 2A and 2B, a significant increase in cell migration was observed in QBC939 and Mz-chA-1 cells after being cultured with MSC(TI)-CM in comparison with control groups.

To confirm the above *in vitro* cell lines results in *in vivo* mice studies, we established an xenograft model in which QBC939 (2 × 10^6^ cell/mice), QBC939 mixed with MSCs (3:1) and QBC939 mixed with MSCs (pretreated with TNF-α and IFN-γ) injected subcutaneously into immunocompromised mice. The growth kinetics of the MSCs containing tumors were compared to those of QBC939 injected alone over the subsequent 1–4 weeks, after that the histopathology of the resulting tumors was studied. We found that both MSCs and pretreated MSCs could accelerate tumor growth (Figure 2C and 2D), while no significance was found between these two groups. These observations also validated our previous results that MSCs can promote CCA growth.

CCA is more prone to liver metastases than other organs [2]. We found that there were varying degrees of liver metastasis in our nude mouse model (Figure 2E, Supplementary Figure 1A). The tumor nodules of the liver were counted, and the results were shown in Figure 2F. MSCs treated with TNF-α and IFN-γ group displayed a marked increase in the number of liver metastases (*P <* 0.05). Figure 2G showed the H&E staining results of metastatic liver, the pathological structure of the control group was just like the normal tissue, and only little part of it has metastatic focus, while the MSCs group and inflammatory cytokines treated MSCs group showed more and larger metastatic focus. These data suggest that MSCs enhance *in vivo* tumor growth and liver metastases, and inflammatory cytokines treated MSCs could increase CCA cells migration and metastases to a greater degree than untreated MSCs.

### MSC(TI)-CM increased the expression of metastatic markers in CCA cell lines

**Figure 3:**
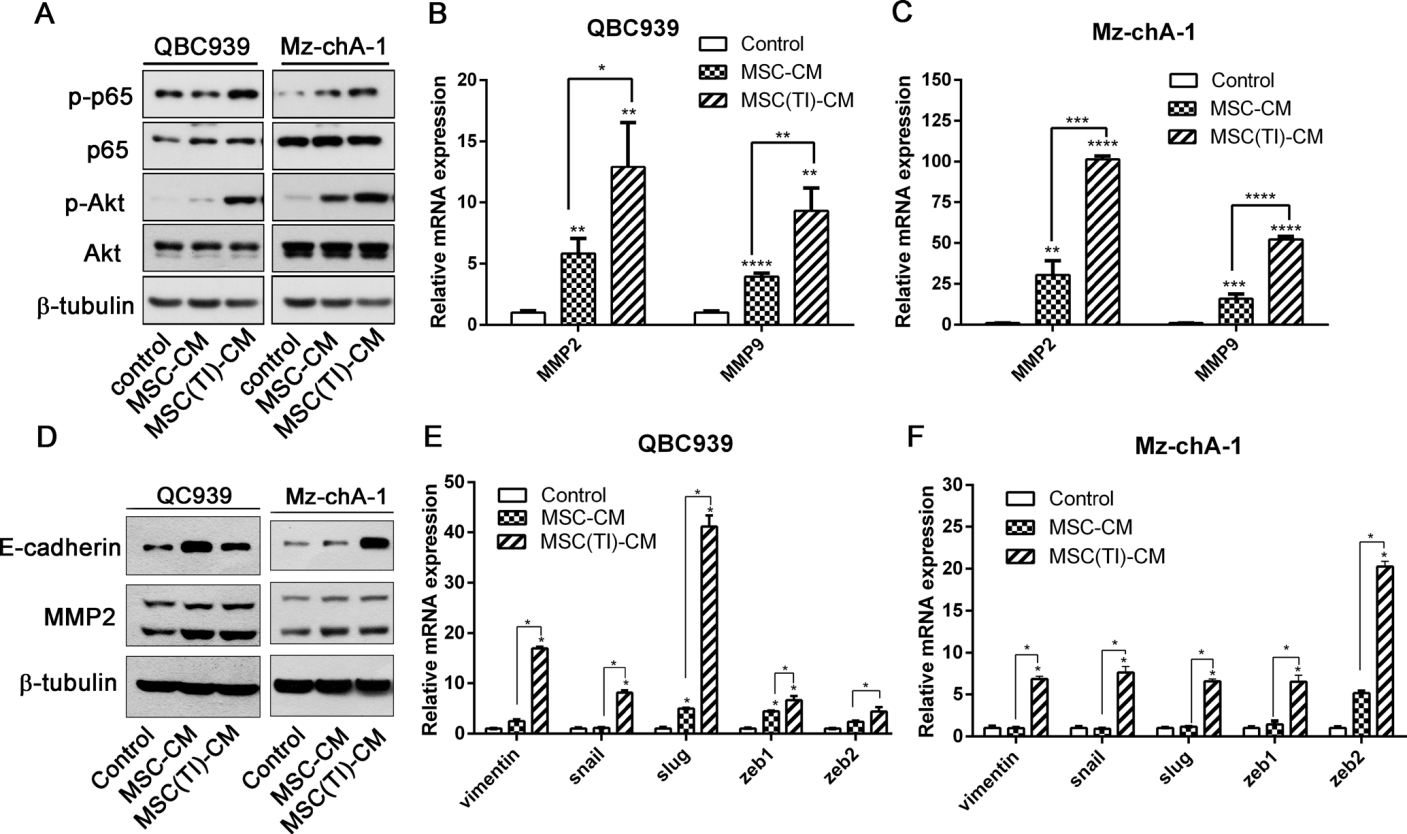
MSC(TI)-CM increased the expression of metastatic markers in CCA cell lines QBC939 and Mz-chA-1 were cultured with MSC-CM or MSC(TI)-CM. The expression or transcription of metastatic markers were analyzed. (**A**) The expression of phosphorylated NF-κB and Akt were analyzed by western blot. (**B**) and (**C**) The transcription of *MMP2* and *MMP9* were analyzed by real-time qPCR. (**D**) QBC939 and Mz-chA-1 were cultured with MSC-CM or MSC(TI)-CM for 72 hours, then the expression of E-cadherin and MMP2 were analyzed by western blot. (**E**) and (**F**) The transcription of EMT markers were tested by real-time qPCR. *GAPDH* mRNA was used to normalize variability in template loading. Date were expressed as fold change (means ± S.D.) over controls, *P < 0.05, **P < 0.01, ***P < 0.001, ****P < 0.0001. Abbreviations: EMT, epithelial-mesenchymal transition; GAPDH, glyceraldehyde-3-phosphate dehydrogenase; ZEB1, zinc-finger E-box binding homeobox 1; ZEB2, zinc-finger E-box binding homeobox 2; MMP2, Matrix Metalloproteinase-2; MMP9, Matrix Metalloproteinase-9.

QBC939 and Mz-chA-1 cells were treated with MSC-CM or MSC(TI)-CM for 24 h, and the total cell protein were analyzed by western blot. As shown in Figure 3A, MSC(TI)-CM increased the phosphorylation of p65 at ser536 and phosphorylation of Akt at ser473 (Figure 3A). The NF-κB and AKT signaling were all activated by MSC(TI)-CM. In addition, the mRNA level of *MMP2* and *MMP9* and the expression of MMP2 were also up-regulated (Figure 3B–3D). To understand whether MSC(TI)-CM culture could cause EMT transition in CCA cell lines, we further detected the mRNA level and protein expression level of some EMT-associated markers. As shown in figure 3D and 3E, MSC-CM induced the transcription of *Vimentin, slug, ZEB1, ZEB2* in QBC939, while had no obvious effects on Mz-chA-1. MSC(TI)-CM showed a marked effects on *Vimentin, snail, slug, ZEB1, ZEB2* mRNA transcription. However, we failed to detect the down-regulation of E-cadherin by both real time PCR assay (data not shown) and western blotting assay (Figure 3D).

### Pro-inflammatory cytokines lead to up-regulation of CCL5 in MSCs

**Figure 4:**
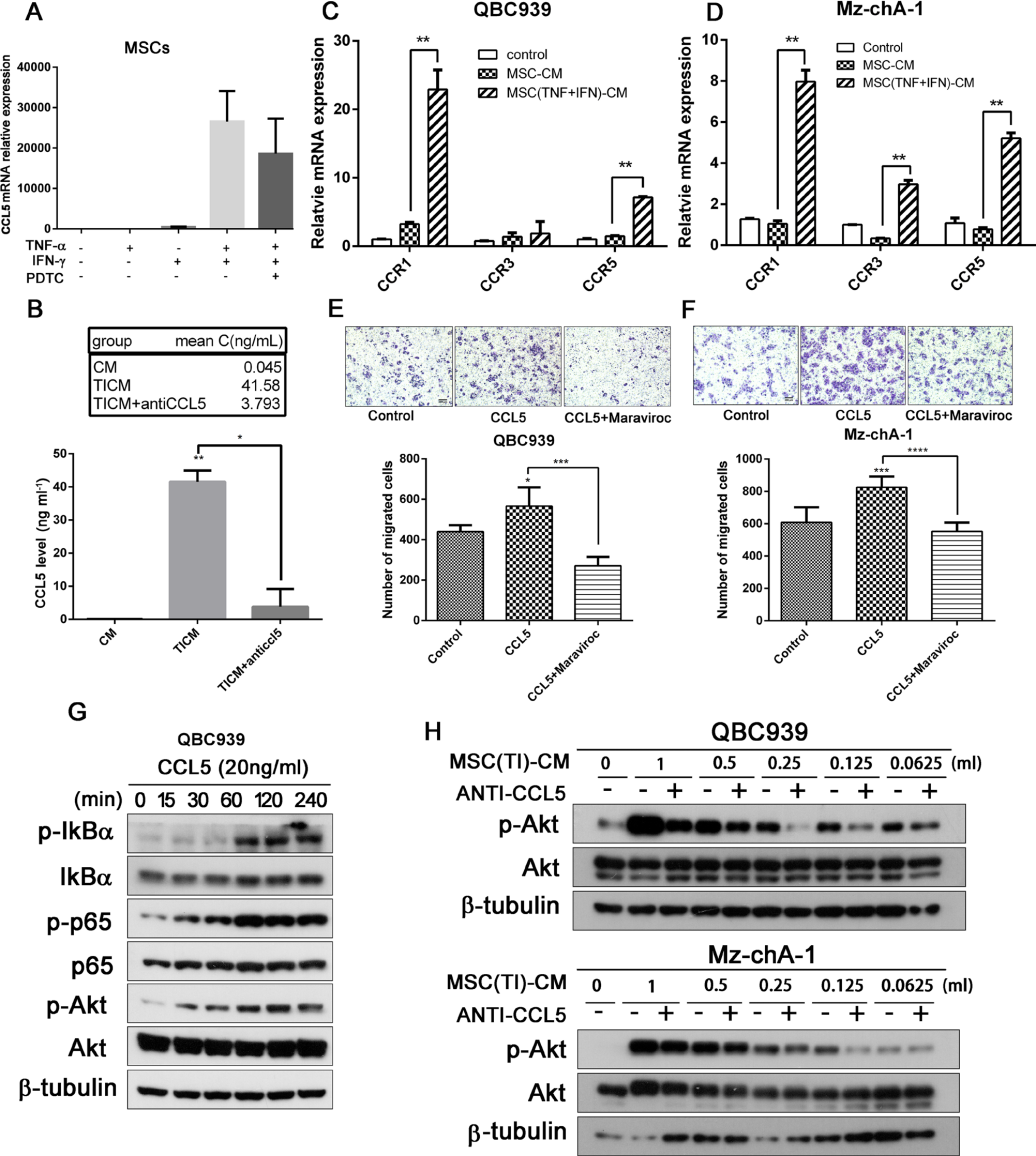
Pro-inflammatory cytokines lead to up-regulation of CCL5 in MSCs **(A)** MSCs were treated with TNF-α and IFN-γ for 6 hours, then the transcription of *CCL5* was analyzed by real-time qPCR. (**B**) MSCs were treated with TNF-α and IFN-γ for 12 hours, then washed with PBS and add fresh medium. 24 h later medium was collected and the secretion of CCL5 was measeured by ELISA. QBC939 (**C**) and Mz-chA-1 (**D**) were cultured with MSC-CM or MSC(TI)-CM for 24 hours, then the total RNA were extracted and the transcription of *CCR1*, *CCR3* and *CCR5* were analyzed by real-time qPCR. (**E**–**F**) CCL5 increased the migration ability of CCA cells QBC939 (E) and Mz-chA-1 (F). Cancer cells were seeded into the up chamber, and CCL5 or CCL5 along with Maraviroc (20 µM) were added into the lower chamber. 24 hours later the migrated cells were stained with crystal violet. (**G**) QBC939 Cells were treated with CCL5 (20 ng/ml) for indicated time intervals and the expression of p-IkB-α, p-p65 and p-Akt in QBC939 cells were analyzed by western blotting. (**H**) QBC939 and M-chA-1 were treated with different concentration of MSC(TI)-CM for 4 hours and the expression of p-Akt; CCL5 neutralizing antibody (0.5 mg/ml) was added to each concentration group to block CCL5. Data are expressed as means±SD, *P < 0.05, **P < 0.01, ***P < 0.001, ****P < 0.0001. Abbreviations: CCL5, chemokine (C-C motif) ligand 5; CCR1, C-C chemokine receptor type 1; CCR3, C-C chemokine receptor type 3; CCR5, C-C chemokine receptor type 5.

We detected the cytokines expression level of MSCs treated by TNF-α and IFN-γ using real-time qPCR assay, and found CCL5 increased markedly (Figures 1F, 4A). CCL5 can be secreted by MSCs, then enhance breast cancer motility, invasion and metastasis [13]. Therefore, we chose CCL5 for further investigation. We proposed hypothesis that CCL5, secreted by MSCs pretreated with TNF-α and IFN-γ, may play an important role in CCA cell invasion and metastasis. The expression of CCL5 was measured by ELISA assay (Figure 4B) and the results were consistent with those of the real-time PCR analysis, a significant (*P <* 0.05) increase of the release of CCL5 was observed in inflammatory cytokines treated MSCs. At the same time, the release of CCL5 could be effectively blocked by CCL5 neutralizing antibodies.

CCL5 activity is mediated through its binding to CCR1, CCR3, and mainly CCR5 [21]. We detected the CCR1, CCR3 and CCR5 transcription in QBC939 and Mz-chA-1 using real-time PCR assay (Figure 4C, 4D). When treated with MSC(TI)-CM, CCR5 increased 4.89 fold in QBC939 and 6.69 fold in Mz-chA-1 compared with MSC-CM group (*P <* 0.05). Then a migration assay was performed to investigate whether MSCs promoting cholangiocarcinoma cell migration and invasion was associated with high expression level of CCL5. The results were presented in Figure 4E and 4F, when treated with MSC(TI)-CM, the number of cancer cells migrating through the membrane increased significantly compared with the control group or MSC-CM group. CCL5 has a similar effect on cancer cell migration, while Maraviroc (20 µM) has an opposite effect (*P <* 0.05). These results indicated that CCL5 may promote CCA cell migration through interact with CCR5.

It has been reported that CCL5 could acts through PI3K/Akt, which in turn activates IKKα/β and NF-κB, and contributing to the migration of human lung cancer cells [24], and NF-κB activation could promote the expression of MMP9 [25]. Hence, we treated QBC939 with CCL5 at different time point and measured the phosphorylation of Akt by western blotting. It can be seen from Figure 4G, CCL5 have the ability to mediate Akt and P65 phosphorylation. Then we cultured cancer cell with different concentration of MSC(TI)-CM along with CCL5 neutralizing antibody (0.5 μg/ml). The phosphorylation of Akt was down-regulated to some extent by blocking CCL5 (Figure 4H). These results informed that proinflammatory cytokines TNF-α and IFN-γ increased the expression of CCL5; CCL5 may have promoting effects on CCA cells migration.

### Akt/NF-κB signaling pathways are involved in CCL5-mediated migration activity

**Figure 5:**
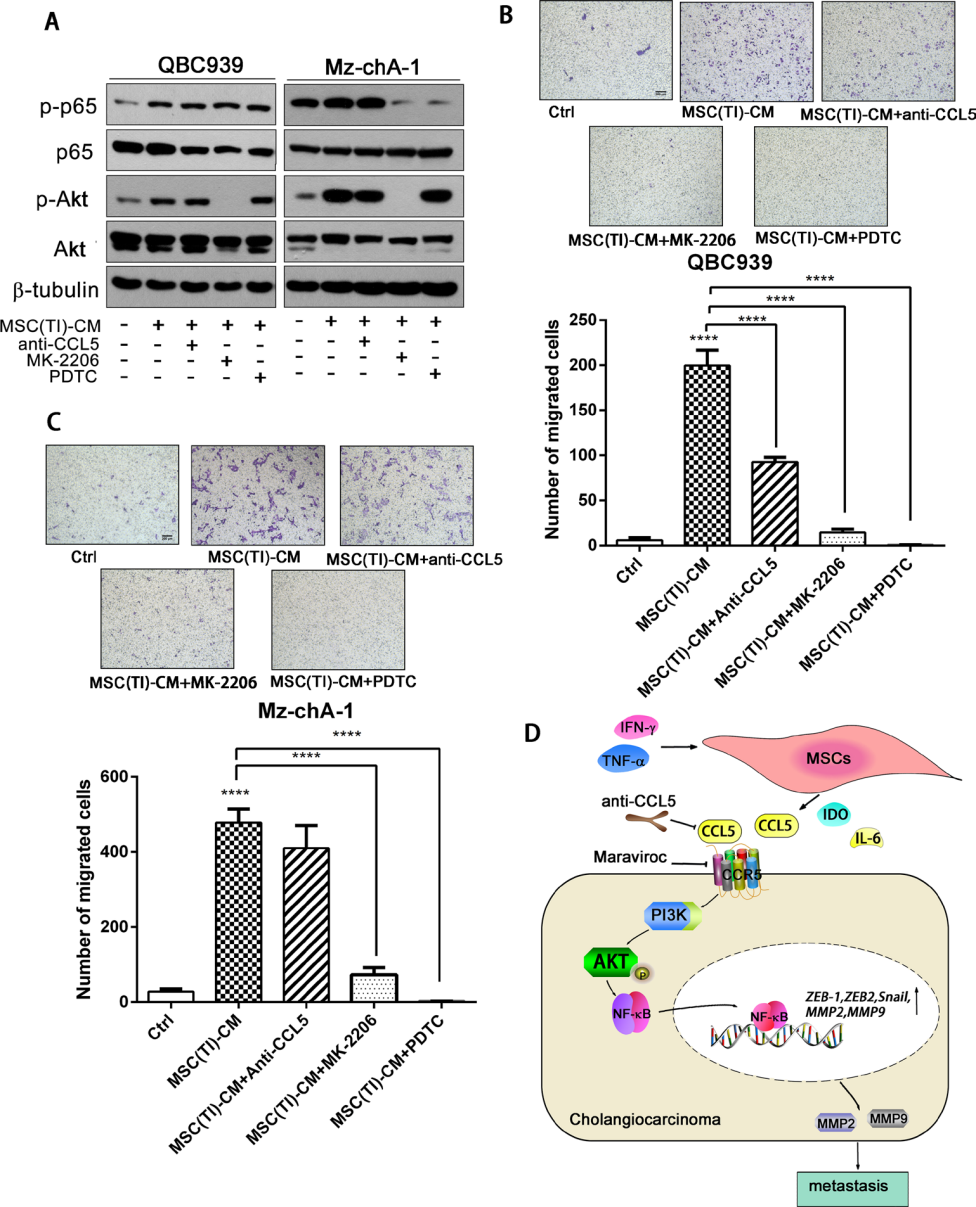
CCL5 secreted by MSCs under inflammatory condition promoted CCA cells migration **(A)** QBC939 and Mz-chA-1 were treated with CCL5 neutralizing antibody, MK-2206 (an Akt inhibitor) or PDTC for an hour, then cultured with MSC(TI)-CM for 4 hours. p-p65 and p-Akt were analyzed by western blotting assay. QBC939 (**B**) and Mz-chA-1 (**C**) were seeded into the up chamber, and MSC(TI)-CM was added into the lower chamber , in the presence or absence of CCL5 neutralizing antibody (0.5 mg/ml), MK-2206 (10 µM) or PDTC (10 µM), 24 hours later the migrated cells were stained and counted. (**D**) A cartoon summarize the results. Data are expressed as means ± SD. *P < 0.05, **P < 0.01, ***P < 0.001, ****P < 0.0001.

Next, we treated QBC939 and Mz-chA-1 with different MSCs conditioned medium, and detected the Akt phosphorylation by western blotting (Figure 5A). MSC-CM and MSC(TI)-CM increased Akt and NF-κB phosphorylation. MK-2206, an inhibitor of Akt, can effectively inhibit the phosphorylation of Akt in MSC(TI)-CM cultured cancer cells. After treated with MK-2206 (10 µM), the phosphorylation of P65 also decreased in Mz-chA-1 cells. However, PDTC just inhibit the phosphorylation of P65 and have no effects on Akt phosphorylation. We further validated these results using migration assay (Figure 5B and 5C). These results suggested that inflammatory environment could increase secretion of CCL5 in MSCs, and CCL5 may interact with CCR5 then mediate Akt/NF-κB phosphorylation. Activated Akt/NF-κB signaling pathway increased the expression of downstream proteins, such as MMP2 and MMP9, and finally result in cancer cell migration, invasion and metastasis (Figure 5D).

## DISCUSSION

Recent researches reported that MSCs have good treatment effects and broad prospect of clinical application in diabetes, hepatic sclerosis, spinal cord injury, wound repair, coronary heart disease and cancer treatments. To clarify the effects of secreted factors of MSCs and avoid inappropriate use of MSCs in clinical therapy, we must fully understand the role of the inflammatory microenvironment on MSCs. As we know, MSCs can be recruited to inflammatory injury and tumor sites playing the tissue repair, immunomodulatory effect, regulating tumor growth and metastasis. There are various inflammatory cytokines gathered in the tumor and inflammatory microenvironment. Owing to these cytokines, MSCs may gain super functions and different characteristics. Under regular culture conditions, bone marrow derived murine MSCs constitutively expressed low levels of COX-2, PGE-2, TGF-β1 and HGF, while treated with inflammatory cytokines TNF-α and IFN-γ, MSCs expressed high level of PGE-2, IDO and PD-L1 [26]. Inflammatory environment make MSCs acquire strong immune modulating function, may promote the tumor immune escape, and tumor invasion. MSCs stimulated by inflammatory cytokines IFN-α and TNF-γ express higher level of VEGF in MSCs, these MSCs then enhance tumor angiogenesis and finally leading to colon cancer growth in mice [11]. MSCs under inflammatory environment condition could promote hepatocellular carcinoma metastasis through TGF-β-induced EMT [27]. Here, we use TNF-α and IFN-γ to simulate inflammatory condition to stimulate MSCs, and found that *TNF-α, IL-6, CCL5, IDO* transcription were increased, while *TGF-β* and *CXCL12* were suppressed (Figure 1F). Inflammatory environment increased MSCs migration ability and activated NF-κB signaling of these MSCs cells. Inflammatory condition played an important role on MSCs phenotype and function.

CCA is rising from cholangiocyte differentiation and with insidious early and atypical clinical symptom, rapid progression, high malignant degree and poor prognosis. Chronic inflammation is an important risk factor to induce bile duct epithelial cells malignant transformation. MSCs could recruited to CCA sites, and its phenotype and functions changed because of inflammatory cytokines in the tumor environments. We performed *in vitro* and *in vivo* studies using both MSCs and TNF-α and IFN-γ treated MSCs. These results informed us that inflammatory environment increased the potency of MSCs to improve CCA cells migration, invasion and metastasis. The effects of MSCs probably mediated by secreted cytokines through paracrine fashion. We also observed that the conditioned medium from inflammatory cytokines treated MSCs have the ability to induce the transcription of *Vimentin*, *snail*, *slug*, *ZEB1* and *ZEB2*. *snail*, *slug*, *ZEB1* and *ZEB2* are master transcription factors, the functions of which are finely regulated at the transcriptional, translational and post-translational levels of EMT [28]. However, we failed to detect the downregulation of E-cadherin (Figure 3D), which was an important marker of EMT. Jing et al. reported that in the inflammatory environment, MSCs expressed high level of TGF-β, and TGF-β can induce EMT progress [28]. Additionally, Li et al. indicated that MSCs could promote hepatocellular carcinoma proliferation but reduced the invasion and metastasis, possibly through down-regulation of TGFβ1 [29]. Our results showed that MSCs under inflammatory condition expressed low level of TGF-β (Figure 1C), this may be one of the reasons of failed to induce CCA EMT.

The function of CCL5 on cancer cell is not well established. CCL5 may have effects on cancer cell proliferation, metastasis, and the formation of an immunosuppressive microenvironment; it may be a potential therapeutic target in several cancer diseases [20]. Karnoub et al. proved that bone-marrow-derived human mesenchymal stem cells could cause the breast cancer cells to increase their metastatic potency, and CCL5 secreted by MSCs acts in a paracrine fashion on the cancer cells to enhance their motility, invasion and metastasis [13]. After infiltrated into the PCa cells, MSCs secreted CCL5, then led to the upregulation of MMP9, ZEB-1, CD133 and CXCR4, and enhanced the metastatic ability of PCa cells [30]. In our present study, MSCs under inflammatory condition exhibited higher expression levels of CCL5 than control MSCs cells, and co-culture of cholangiocarcinoma cells with MSC(TI)-CM induced upregulation of CCR1, CCR3 and CCR5 expression. CCL5 receptors include CCR1, CCR3 and CCR5, while in cancer research CCR5 is the main receptor. Therefore, we chose CCR5 for further investigation. Maraviroc is an antagonist of CCR5, it can prevents development of hepatocellular carcinoma in a mouse model [31]. We used Maraviroc or anti-CCL5 antibody to block the CCL5 in the conditioned medium. The results showed that both of them could effectively inhibit Mz-chA-1 cell migration (Figure 5B). These results indicated that inflammatory environment increased CCL5 expression and secretion in MSCs, and then cholangiocarcinoma cell invasion was increased by CCL5/CCR5 axis.

CCR5 is a G-protein-coupled receptor that can mediate various signaling cascade. There are a number of cancer expressed CCR5 but only a part of them are widely studied. Over-expressed CCL5 in MDA-MB-231 exhibited higher levels of the Ser 473-phosphorylated, activated form of Akt [13]. CCL5 stimulation increased phosphorylation of Akt, activated NF-κB signal pathway, promoting human lung cancer migration [24]. CCL5 can also promoting angiogenesis in human CCA cells by down-regulating miR-200b through PI3K/Akt signaling pathway [32]. However, seldom research has been done in CCA. We hypothesized that CCL5 could activate Akt signaling pathway partly through CCR5, and then promoting cancer cell invasion. We stimulated QBC939 with recombinant CCL5, and verified that CCL5 have the ability to induce Akt phosphorylation and increase the migration ability of CCA cells. We also detected that inflammatory treated MSCs could activated Akt signal pathway (Figure 3A), and increase the transcription and expression of MMP2 and MMP9. Thus, these results indicated that CCL5/CCR5 axis could activated Akt signal pathway, then activated the downstream signaling, and promoting the expression of MMP2 and MMP9, increase the cholangiocarcinoma cell invasion and metastasis.

MSCs can secrete large amount of cytokines. The mechanism of MSCs effects on cancer cell progression via paracrine fashion is complex. CCL5 may play a crucial effect on CCA cells invasion and metastasis, but there may be other factors also influenced on cancer cell invasion and metastasis. MSCs are more likely to influenced cancer cell progression through cytokines network.

In conclusion, our study showed that inflammatory environment could activated NF-kB signal pathway in MSCs, increased the expression and secretion of cytokines and chemokines, such as IL-6, CCL5 and TNF-α. Inflammation also increased MSCs migration ability. Furthermore, we demonstrated the critical role of CCL5/CCR5 in migration of CCA cells induced by MSCs in inflammation microenvironment. CCL5 interact with CCR5, then activate Akt/NF-κB signaling, promoting MMP2 and MMP9 expression, and finally result in CCA cells invasion and metastasis.

## MATERIALS AND METHODS

### Mice and ethics statement

Male BALB/c-nu/nu mice, at age of 4–5 weeks, were purchased from Xiamen University Laboratory Animal Center. All experimental mice were raised under standard conditions (SPF house, 12-hour day-night rhythm). All animal procedures were approved by the Animal Care and Use Committee of Xiamen University (license No: SYXK [Min] 2008-0003, issued on May 6, 2008).

### Cell lines

Human umbilical cord mesenchymal stem cells (hUC-MSCs) were cultured with α-MEM medium (Gibco, Grand Island, NY, USA) supplemented with 10% fetal bovine serum (FBS) (Gibco, Grand Island, NY, USA) and 100 U/mL penicillin/streptomycin. The third to eighth passages of MSCs were used in the following experiments. Human cholangiocarcinoma cell lines QBC939 and Mz-ChA-1(kindly provided by the First affiliated hospital of Xiamen university) were cultured in RPMI 1640 medium (Gibco, Grand Island, NY, USA) supplemented with 10% FBS and 100 U⁄mL penicillin⁄ streptomycin solution. All cells were maintained in a humidified of 95% and 5% CO_2_ environment at 37°C.

### Identification of MSCs

MSCs were obtained from stemcell technologies inc. and characterized by flow cytometric analysis with FITC-CD29, FITC-CD34, FITC-CD44, FITC-CD45 and FITC-CD90 antibodies (ebiociece, San Diego, USA). Cells were cultured and harvested after they grew to 80% of the dish. After been washed with phosphate buffered saline (PBS) twice, MSCs were stained with antibodies against CD29, CD34, CD44, CD45 and CD90, and IgG1 or IgG2b added as the isotype control. Samples were analyzed by Flow cytometry (Becton Dickinson, Franklin Lakes, NJ, USA). For adipogenic and Osteogenic differentiation assay, Human Umbilical Cord MSC Adipogenic Differentiation Medium and Osteogenic differentiation Medium (cyagen, Guangzhou, China) were used according to the instructions.

### Preparation of MSCs conditioned media (MSC-CM)

hUC-MSCs were cultured to 90% confluence. Washed with PBS twice, treated with TNF-α (20 ng/ml) and IFN-γ (50 ng/ml) for 12 hours, then washed with PBS twice and add serum-free RPMI-1640 to each dish for another 24 hours. The conditioned media were filtered through the 0.22 μm pore sterile filter and stored at –80°C for further use within one week.

### Cell proliferation assay

For proliferation assay, cholangiocarcinoma cells were planted in 96 well dished and allowed to attach overnight. Then cells were treated with TNF-α and/or IFN-γ for 0 to 48 h. 20 μl 3-(4,5-dimethylthiazol-2-yl)-2,5-diphenyltetrazolium bromide (MTT) (5 mg/ml) were add to each well for the last 4 h. At the end of the experiment, all the solution was discarded and 150 μl of dimethyl sulfoxide (DMSO) was added to each well. The 96-well plate was shaken to ensure complete solubilization of the purple formazan crystals. Absorbance at 490 nm was measured by an enzyme-linked immunosorbent assay reader.

### Transwell migration assay

The cholangiocarcinoma cells (10^5^–5 × 10^5^ cells) were added into the upper chamber with 100 µl serum free medium, MSC-CM and MSC(TI)-CM alone or combined with Maraviroc (Sigma Chemical Co, MO,USA) (20 µM), MK-2206 (Selleck Chemicals, TX, USA) (10 µM), anti-CCL5 antibody (PeproTech, NJ, USA) (0.5 µg/ml) or PDTC (Selleck Chemicals, TX, USA) (10 µM) were added into the lower chamber (containing 10% FBS). 24 hours later, remove the cell which were not migrated to the the reverse side of the filters and washed with PBS. Then 4% paraformaldehyde fixed the migration cells for 10 minutes, stained with crystal violet staining, and counted in five non-overlapping fields under a bright-field microscopy (IX51, Olympus Corporation, JPN) at 100 magnification.

### Real-time reverse transcriptase-PCR

Total cellular RNA was prepared using TRNzol reagent (Tiangen, Beijing, CHN) and the expression levels of *TNF-a, IL-6, CXCL12, TGF-β, CCL5, E-cadherin*, *Vimentin*, *N-cadherin*, *Snai1*, *Slug*, *ZEB1* and *ZEB2* mRNA were determined by real-time reverse transcriptase–PCR using GoTaq^®^ qPCR Master Mix (Promega, Madison, WI, USA). Data were normalized to GAPDH expression and represent the average of three repeated experiments. The primers for specific genes were shown in Supplementary Table 1.

### Western blotting

Western blotting was performed as previous described [33, 34]. In short, Samples were collected by lysing cells in RIPA lysis buffer. Each sample was size-fractionated using SDS-polyacrylamide gel electrophoresis (PAGE) and electrotransferred onto polyvinylidene difluoride transfer membranes (Dupont, Boston, MA, U.S.A.).After bolted with milk, the membranes incubated with primary antibodies overnight at 4°C, and then blotted with horseradish peroxidase conjugated secondary antibodies. The immunoblots were visualized using ECL (GE Healthcare, Bucks, UK).

### *In vivo* assays of tumor growth and metastasis in nude mice bearing human CCA

QBC939 cells and MSCs were prepared either as single-cell type suspensions (2 × 10^6^ cells in 100 µl of PBS) or as mixtures of cells (2 × 10^6^ QBC939 cells and 7 × 10^5^ MSCs in 100 µl of PBS). Subcutaneous administration of QBC939 cells (alone or mixed with MSCs) was performed in the armpit areas of Balb/c mice. Mice were examined every 3 days and tumor growth was evaluated by measuring the length and width of the tumor mass. Tumor volume was estimated according to the formula: volume (mm^3^) = 0.5 a^2^·b. On the 30th day after implantation, the mice were killed and the tumors were removed and preserved for the following investigations. Liver metastases were evaluated by hematoxylin and eosin (H&E) staining and tumor nodules were counted by macroscopic observation.

### H&E staining

Liver tissues were fixed in 4% buffered formaldehyde, then dehydrated, and subsequently embedded in paraffin. Tissue sections were cut at a thickness of 5 µm and dewaxed, then the slides were rinsed in phosphate-buffered saline and stained with hematoxylin and eosin.

### Immunoassay for CCL5

The secretion level of CCL5 in the MSC-CM and MSC(TI)-CM were assessed by Human RANTES ELISA Kit (BOSTER, Wu Han, CHN) and test using a Model 680 microplate reader (Bio-Rad Life Sciences) according to the manufacturer’s protocols.

### Statistical analysis

Data were presented as the means±S.D. for at least three separate determinations of each group. The differences between the groups were examined for statistical significance using the Student’s *t*-test with GraphPad Prism 6.0 software. Differences were considered significant when the *p* < 0.05.

## SUPPLEMENTARY MATERIALS FIGURE AND TABLE


