# Supplementary material for: Mesenchymal stem cells in inflammatory microenvironment potently promote metastatic growth of cholangiocarcinoma via activating Akt/NF-κB signaling by paracrine CCL5
Source: Oncotarget. 2017 May 11;8(43):73693–704. doi: 10.18632/oncotarget.17793 (PMC5650292; doi:10.18632/oncotarget.17793)
Supplement: Supplementary file 1 [file oncotarget-08-73693-s001.pdf]

Mesenchymal stem cells in inflammatory microenvironment potently promote metastatic growth of cholangiocarcinoma *via* activating Akt/NF-κB signaling by paracrine CCL5

SUPPLEMENTARY MATERIALS

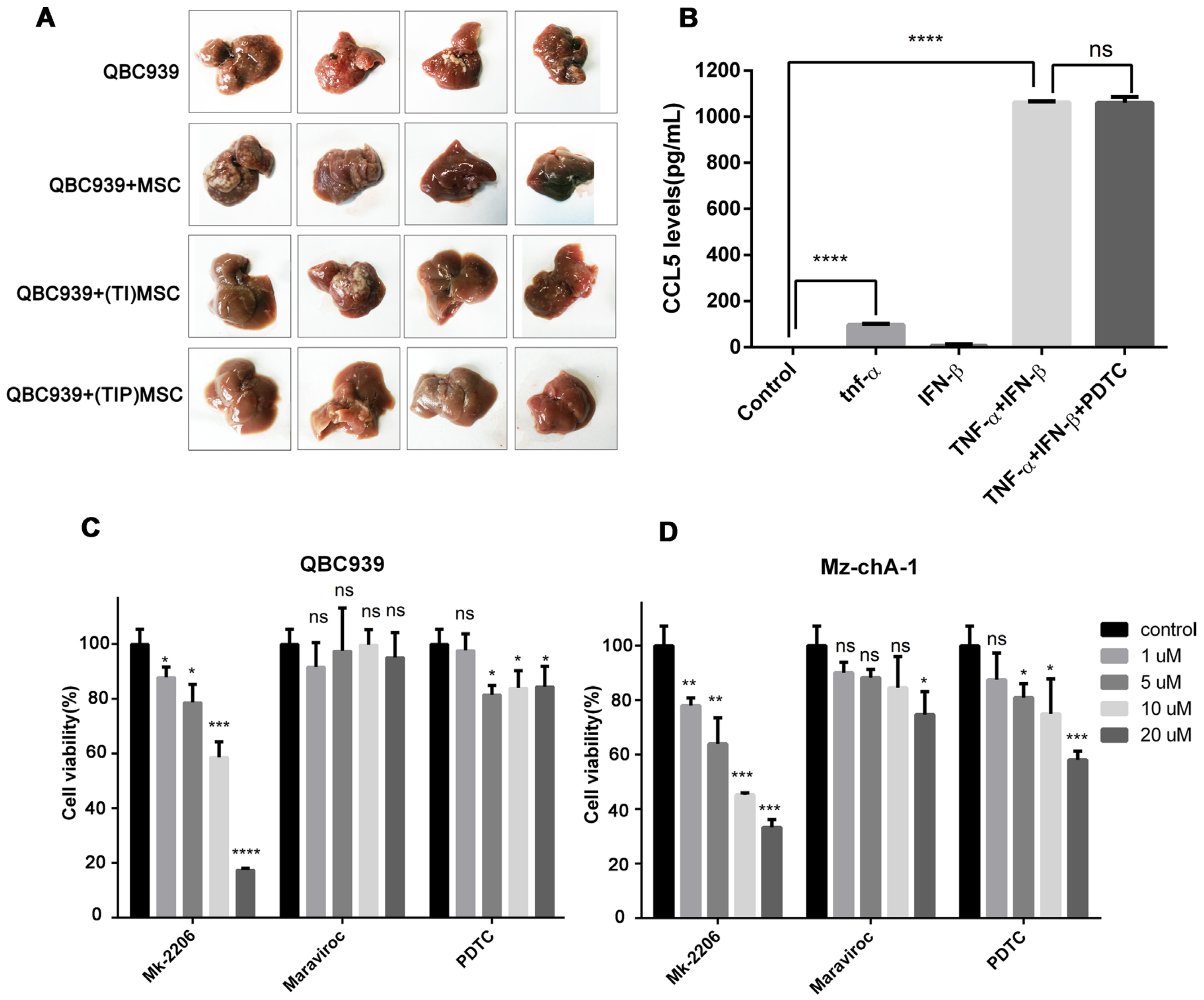

Supplementary Figure 1: (**A**) Picture showing metastasized tumor nodules in liver tissue. (**B**) PDTC can not inhibit the expression of CCL5 which induced by TNF-α and IFN-γ. (**C–D**) Effects of MK-2206, Maraviroc and PDTC on cell viability of QBC939 (C) and Mz-chA-1 (D). Data are presented as means ± SD. $^{*}P < 0.05$, $^{**}P < 0.01$, $^{***}P < 0.001$, $^{****}P < 0.0001$; two-tailed Student's *t* test.

Supplementary Table 1: Real-time RT-PCR primers

Gene	Forward primer(5′–3′)	Reverse primer(5′–3′)
IL-6	GGCACTGGCAGAAAACAACC	GCAAGTCTCCTCATTGAATCC
TNF-a	GGCGTGGAGCTGAGAGATAAC	GGTGTGGGTGAGGAGCACAT
CCL5	TACCATGAAGGTCTCCGC	GACAAAGACGACTGCTGG
TGF-b	CCCACAACGAAATCTATGAC	CCAGGAATTGTTGCTGTATT
Periostin	TGTTGCCCTGGTTATATGAG	ACTCGGTGCAAAGTAAGTGA
IDO	GCCCTTCAAGTGTTTCACCAA	CCAGCCAGACAAATATATGCGA
CCR5	GTATCTGGCATAGTATTCTGT	ATCTCTGGTCTGAAGGTT
E-cadherin	TGCCCAGAAAATGAAAAAGG	GTGTATGTGGCAATGCGTTC
Vimentin	GAGAACTTTGCCGTTGAAGC	GCTTCCTGTAGGTGGCAATC
Snail	CTGGGTGCCCTCAAGATGCA	CCGGACATGGCCTTGTAGCA
Slug	TACCGCTGCTCCATTCCACG	CATGGGGGTCTGAAAGCTTGG
ZEB1	TGCACTGAGTGTGGAAAAGC	TGGTGATGCTGAAAGAGACG
ZEB2	CGGTATTGCCAACCCTCTGGA	TTGTTGTGCCAGGGGTGTTCC
GAPDH	TGCACCACCAACTGCTTAGC	GGCATGGACTGTGGTCATGAG
